# Oridonin alleviates the inhibitory effect of lipopolysaccharide on the proliferation and osteogenic potential of periodontal ligament stem cells by inhibiting endoplasmic reticulum stress and NF-κB/NLRP3 inflammasome signaling

**DOI:** 10.1186/s12903-023-02827-0

**Published:** 2023-03-09

**Authors:** Junhao Jiang, Nong Zhang, Haibo Song, Ya Yang, Juan Li, Xiaoli Hu

**Affiliations:** 1 Department of Stomatology, Shenzhen Longgang District Maternity & Child Healthcare Hospital(Longgang Maternity and Child Institute of Shantou University Medical College), Shenzhen, 518172 China; 2 Department of Operative Dentistry and Endodontics, Guanghua School and Hospital of Stomatology, Sun Yat-Sen University, Guangzhou, 510055 Guangdong China

**Keywords:** Oridonin, Endoplasmic reticulum (ER) stress, NF-κB/NLRP3 inflammasome, Human periodontal ligament stem cells (hPDLSCs), Osteogenic potential

## Abstract

**Background:**

The aim of this study was to investigate the protective effect and mechanism of oridonin in an in vitro lipopolysaccharide (LPS)-induced human periodontal ligament stem cells (hPDLSCs) model of periodontitis.

**Methods:**

Primary hPDLSCs were isolated and cultured, and then the expression of surface antigens CD146, STRO-1 and CD45 of hPDLSCs was detected by flow cytometry. The mRNA expression level of Runx2, OPN, Col-1, GRP78, CHOP, ATF4 and ATF6 in the cells was tested by qRT-PCR. MTT was taken to determine the cytotoxicity of oridonin at different concentrations (0–4 μM) on hPDLSCs. Besides, ALP staining, alizarin red staining and Oil Red O staining were utilized to assess the osteogenic differentiation (ALP concentration, mineralized calcium nodule formation) and adipogenic differentiation abilities of the cells. The proinflammatory factors level in the cells was measured by ELISA. The protein expression level of NF-κB/NLRP3 pathway-related proteins and endoplasmic reticulum (ER) stress-related markers in the cells were detected by Western blot.

**Results:**

hPDLSCs with positive CD146 and STRO-1 expression and negative CD45 expression were successfully isolated in this study. 0.1–2 μM of oridonin had no significant cytotoxicity on the growth of hPDLSCs, while 2 μM of oridonin could not only greatly reduce the inhibitory effect of LPS on the proliferation and osteogenic differentiation of hPDLSCs cells, but also inhibit LPS-induced inflammation and ER stress in hPDLSCs cells. Moreover, further mechanism research showed that 2 μM of oridonin suppressed NF-κB/NLRP3 signaling pathway activity in LPS-induced hPDLSCs cells.

**Conclusions:**

Oridonin promotes proliferation and osteogenic differentiation of LPS-induced hPDLSCs in an inflammatory environment, possibly by inhibiting ER stress and NF-κB/NLRP3 pathway. Oridonin may have a potential role in the repair and regeneration of hPDLSCs.

**Supplementary Information:**

The online version contains supplementary material available at 10.1186/s12903-023-02827-0.

## Background

Periodontitis is a common chronic inflammatory disease of tooth loss caused by periodontal supporting tissue destruction with a high incidence [1]. With the progression of periodontitis, patients experience tooth mobility, displacement or even loss. These symptoms have a serious impact on patients' mastication, pronunciation and their oral health and life, and even lead to tooth unusable. Although the commonly used treatments can control the progression of inflammation and prevent further alveolar bone resorption, they cannot effectively repair the alveolar bone defects that have been caused [2]. Fortunately, stem cell therapy and tissue engineering methods with seed cells as the core provide a new therapeutic strategy for defect tissue repair [3].

Periodontal ligament stem cells (PDLSCs) are mesenchymal stem cells (MSCs) with multilineage differentiation potential in the periodontal ligament cell population with proliferation and anti-apoptosis abilities. Besides, PDLSCs are considered to be seed cells with most potential for periodontal and bone tissue regeneration [4, 5]. Nevertheless, due to the insufficient number of stem cells and the influence of inflammation, the periodontal tissue regeneration ability often cannot meet the needs of treatment in the process of periodontal tissue repair [6]. Endoplasmic reticulum (ER) stress was found to participate in reducing the osteogenic differentiation ability of PDLSCs in the inflamed microenvironment induced by tumor necrosis factor-α (TNF-α), which indicated ER stress could promote the damage of periodontal tissue in periodontitis [7]. What’s more, NLRP3 inflammasome also plays an important role in intracellular inflammation and osteogenic differentiation of PDLSCs, triggering production of inflammatory cytokines typically via Toll or other receptors that activate the NF-κB signaling pathway [8, 9]. Soundara et al. [10] found that the anti-inflammatory role of hPDLSCs and their secretory products may be due to the suppression of NF-κB stimulation, which in turn inhibited NALP3 inflammasome activation.

Oridonin is a natural terpenoid isolated from traditional Chinese herbal medicine *Isodon rubescens* (Hemsl.), with a wide range of pharmacological properties, including anti-cancer, anti-inflammatory, hepatorenal activities as well as cardioprotective activities and so on [11]. For example, a number of previous studies have indicated that oridonin exerts an essential anticancer effect in cancers such as colorectal cancer [12], nasopharyngeal carcinoma [13], and acute myelogenous leukemia [14]. In addition, oridonin also plays a protective role in diseases such as acute liver injury [15], irritable bowel syndrome after inflammation [16], and cardiac hypertrophy [17]. He et al. have revealed that oridonin is a covalent inhibitor of NLRP3 inflammasome, exerts preventive or therapeutic effects on mouse models of peritonitis, gouty arthritis, and type 2 diabetes by inhibiting the activation of NLRP3 inflammasome, thus further effectively inhibiting NLRP3-related diseases [18]. It has been demonstrated that oridonin prolonged the survival rate of mouse cardiac allografts by inhibiting the NF-κB/NLRP3 pathway [19]. However, the role of oridonin in periodontitis has not been reported, so we constructed a in vitro model of hPDLSCs by LPS induction. The aim was to excavate the effects of oridonin on proliferation, osteogenic differentiation inhibition, inflammation and endoplasmic reticulum (ER) stress in LPS-induced hPDLSCs through a series of molecular and cell biology experiments, so as to offer a theoretical basis for the clinical treatment of periodontitis.

## Materials and methods

### Isolation and groups intervention of primary periodontal ligament stem cells

Healthy premolars donated by periodontally healthy individuals undergoing orthodontic treatment at Shenzhen Longgang District Maternity & Child Healthcare Hospital were selected. Then, periodontal ligament tissue from the tooth’s root was scraped with a sharp knife and collected. Next, the tissue was enzymatically hydrolyzed with 0.3% collagenase type I for 30 min and then centrifuged. Subsequently, the sediment was transferred to a culture flask and cultured in α minimum essential medium (a-MEM) containing 10% fetal bovine serum (FBS, Gibco) and 1% penicillin streptomycin (PS) (Solarbio, China). When reaching a confluence of 70%, the primary PDLSCs were passaged cultured, and the third-generation cells were taken for subsequent tests [20]. In addition, the above teeth were collected with informed consent from donors and the experiment was approved by the Ethics Committee of Shenzhen Longgang District Maternity & Child Healthcare Hospital (LGFYYXLLL-2022-009).hPDLSCs cells in the logarithmic growth phase were taken and seeded in a 6-well plate at the same density. And the cells were divided into four groups. CTRL group: untreated hPDLSCs; Ori group: hPDLSCs were treated with 2 μM oridonin (Ori) 24 h; LPS group: hPDLSCs were treated with 10 μg/mL LPS 24 h [20]; LPS + Ori group: hPDLSCs were treated with 10 μg/mL LPS and 2 μM oridonin for 24 h.

### Flow cytometry

After the removal of the culture medium, the third generation of passage cultured hPDLSCs were collected and washed with PBS. Next, the cells were soaked in PBS buffer containing 0.25% trypsin for 5 min to make a single cell suspension. Then, the cells were washed with PBS solution containing 1% bovine serum albumin, and the cell concentration was adjusted to 1 × 10^6^ cells/mL, followed by incubation with monoclonal antibodies CD146 (Abcam, UK), CD90 (Abcam, UK), STRO-1 (RampD systems, USA) and CD45 (Abcam, UK) for 30 min at 4 °C in the dark. Again, cells were washed with PBS, centrifuged at 1000 g for 5 min and resuspended in PBS. Lastly, the background marker was determined using isotype control monoclonal antibody, and the positive rate (%) of cell surface antigen was analyzed by flow cytometry combined with special supporting software.

### Osteogenesis and adipogenesis induction

Osteogenic differentiation induction was performed on hPDLSCs. When the cell confluence reached about 70%, mineralization induction solution (α-MEM medium containing 10% FBS, 1% PS, 50 ng/mL ascorbic acid, 10 mmol/mL β-glycerophosphate sodium and 4 ng/mL dexamethasone) was added. And the solution was changed every 3 d, ALP staining was performed until the 4th day of culture, and alizarin red staining was conducted on the 14th day to observe the osteogenic differentiation.

Adipogenic differentiation induction was performed on hPDLSCs. After the cells were completely grown, adipogenic induction solution A (α-MEM medium containing 10% FBS, 1% PS, 10 μg/mL insulin, 1 μmol/L indomethacin, and 0.5 mmol/L IBMX) was added. After 3 d, B solution (α-MEM medium containing 10% FBS, 1% PS, and 10 μg/mL insulin) took the place of solution A. One day later, solution A substituted for solution B, while 3 d later, solution B again replaced A. Eventually, oil red O staining was utilized 1 d later for adipogenesis detection.

### ALP staining and alizarin red staining

The early mineralization was determined based on the instructions of BCIP/NBT alkaline phosphatase chromogenic kit (Beyotime, Shanghai, China). Cells were fixed in 4% paraformaldehyde (Beyotime) for 10 min, washed three times with PBS, and then incubated with BCIP/NBT staining solution for 30 min at 37 °C in the dark. After that, the treated cells were washed twice with distilled water and photographed. Then, ALP concentration was quantitatively analyzed. Late mineralization was measured according to the instructions of the Osteoblast Mineralized Nodule Staining Kit (Beyotime). The cells were fixed with 4% paraformaldehyde for 10 min, washed with PBS for another three times, and stained with alizarin red for 10 min. Subsequently, the treated cells were washed with distilled water to be observed and photographed microscopically, and the calcium nodules level was then quantitatively analyzed.

### Oil red O staining

Adipogenic differentiation of hPDLSCs cells was determined following the steps of Oil Red O staining kit (Beyotime). The cells were fixed in 4% paraformaldehyde, washed three times with PBS, and covered with an appropriate amount of staining wash buffer for 20 s. Later, the wash buffer was removed and a proper amount of Oil Red O staining solution was added to carry out cell staining for 10–20 min. Afterwards, Oil Red O staining solution was removed, and the right amount of staining wash buffer was added to stand for 30 s. And then, the buffer was removed and the cells were washed with PBS for 20 s. Lastly, an appropriate amount of PBS was added to evenly cover the cells, and the cells were placed under a microscope for observation and photography.

### MTT cytotoxicity assay

hPDLSCs in logarithmic growth phase were collected, and the cell concentration was adjusted to 5 × 10^4^ cells/mL. There were 100 μL of treated cells added into a 96-well plate, and different concentrations of oridonin were added to make the final concentrations of 0, 0.1, 0.5, 1, 2, 3, and 4 μM, respectively. The treated cells were cultured in a 37 ℃, 5% CO_2_ incubator for 24 h. After that, the proliferation rate of hPDLSCs was determined according to the instructions of MTT kit (Beyotime). Specifically, 10 μL of MTT solution (5 mg/mL) was added to each well for another 4-h cell culture. Next, the supernatant was removed, and 100 μL of Formazan solution was added to each well. The cells were mixed at ambient temperature and continued to culture until the sediment was dissolved. The absorbance value at 570 nm was measured by a microplate reader. Finally, the proliferation rates of hPDLSCs treated with different concentrations of oridonin was calculated. After screening for the optimal concentration of oridonin, then hPDLSCs were treated with LPS and oridonin for 24 h, the proliferation rate of hPDLSCs of different groups was determined as previously described.

### ELISA

After the LPS and oridonin treatment for 24 h, cells supernatants from different groups were collected. The level of tumor necrosis factor-α (TNF-α), interleukin-6 (IL-6) and IL-1β in hPDLSCs cells were measured according to the instructions of the ELISA kit (Nanjing Jiancheng Bioengineering Institute, China).

### Real-time PCR (qRT-PCR)

After the LPS and oridonin treatment for 24 h, hPDLSCs cells were collected and total cellular RNA was extracted using Trizol reagent (Sigma). The concentration and purity of extracted total RNA were determined by Nanodrop (Thermo Scientific, Waltham, MA, USA), and then cDNA was synthesized by reverse transcription according to the reverse transcription-PCR kit (Takara) instructions. Afterwards, the synthesized cDNA was taken to test the mRNA expression levels and β-actin as referring to the real time PCR kit (Takara) instructions. Primer sequences adopted was shown in Table 1. Data analysis was performed with the 2^−ΔΔCt^ method.

**Table 1:** Primer sequences

Genes	Primer sequences (5' to 3')
Runx2	Forward: TCTCTGGTTTTTAAATGGTTA
	Reverse: CTTGTACCCTCTGTTGTAAAT
OPN	Forward: ACCGAGACACCATGAGAG
	Reverse: CTGGGTCTCTTCACTACCT
Col-1	Forward: GAGGGCCAAGACGAAGACATC
	Reverse: CAGATCACGTCATCGCACAAC
GRP78	Forward: GTTTGCTGAGGAAGACAAAAAGCT
	Reverse: CACTTCCATAGAGTTTGCTGATAATTG
CHOP	Forward: CCTGAAAGCAGAAACCGGT
	Reverse: CCTCATACCAGGCTTCCAGC
ATF4	Forward: TATGGATGG GTTGGTCAGTG
	Reverse: CTCATCTGGCATGGT TTCC
ATF6	Forward: GGATTTGATGCCTTGGGAGTCAGAC
	Reverse: ATTTTTTTCTTTGGAGTCAGTCCAT
β-actin	Forward: ATCTGGCACCACACCTTC
	Reverse: AGCCAGGTCCAGACGCA

### Western blot

After the LPS and oridonin treatment for 24 h, cells from each group were collected and lysed on ice for 20 min by adding RIPA lysate (Solarbio). Next, the lysed cells were centrifuged at 10,000 rpm, 4 °C for 15 min to collect proteins and the protein concentrations were detected through a BCA kit (Solarbio). There was 30 μg of protein taken and denatured at 95 °C for 15 min with addition of the protein loading buffer. Later, the protein bands were separated by SDS PAGE electrophoresis, and then the protein was transferred to PVDF membranes and blocked in 5% skimmed milk blocking solution for 1–3 h. Subsequently, the membranes were washed three times in TBST buffer and then incubated overnight at 4 °C on a shaker with the addition of diluted primary antibodies (p65, ab16502, Abcam; p-p65, ab76302, Abcam; NLRP3, ab263899, Abcam; Caspase-1, ab138483, Abcam; GRP78, ab21685, Abcam; CHOP, #5554, CST; ATF4, ab184909, Abcam; ATF6, ab122897, Abcam; β-actin, ab6276, Abcam, UK). Afterwards, the membranes were washed with TBST twice, and diluted secondary antibody (Zhongshan Golden Bridge Biotechnology Co., Ltd., China) was added for 1-h incubation at ambient temperature. Again, the membranes were washed with TBST three times, and ECL hypersensitive luminescence solution (Solarbio) was added dropwise to develop the protein bands in a gel imager for photography. The protein bands were measured in grayscale employing Image J software and finally, the relative expression level of the target protein was analyzed with β-actin as an internal control.

### Statistical analysis

The data results were expressed as mean ± standard deviation (SD), and SPSS 24.0 software was utilized for statistical analysis. Student's t-test was adopted for comparison between two groups, and one-way analysis of variance was applied to pairwise comparison among multiple groups. *P* < 0.05 was used as the judgment criteria for significant difference.

## Results

### Isolation and identification of hPDLSCs

**Fig. 1:**
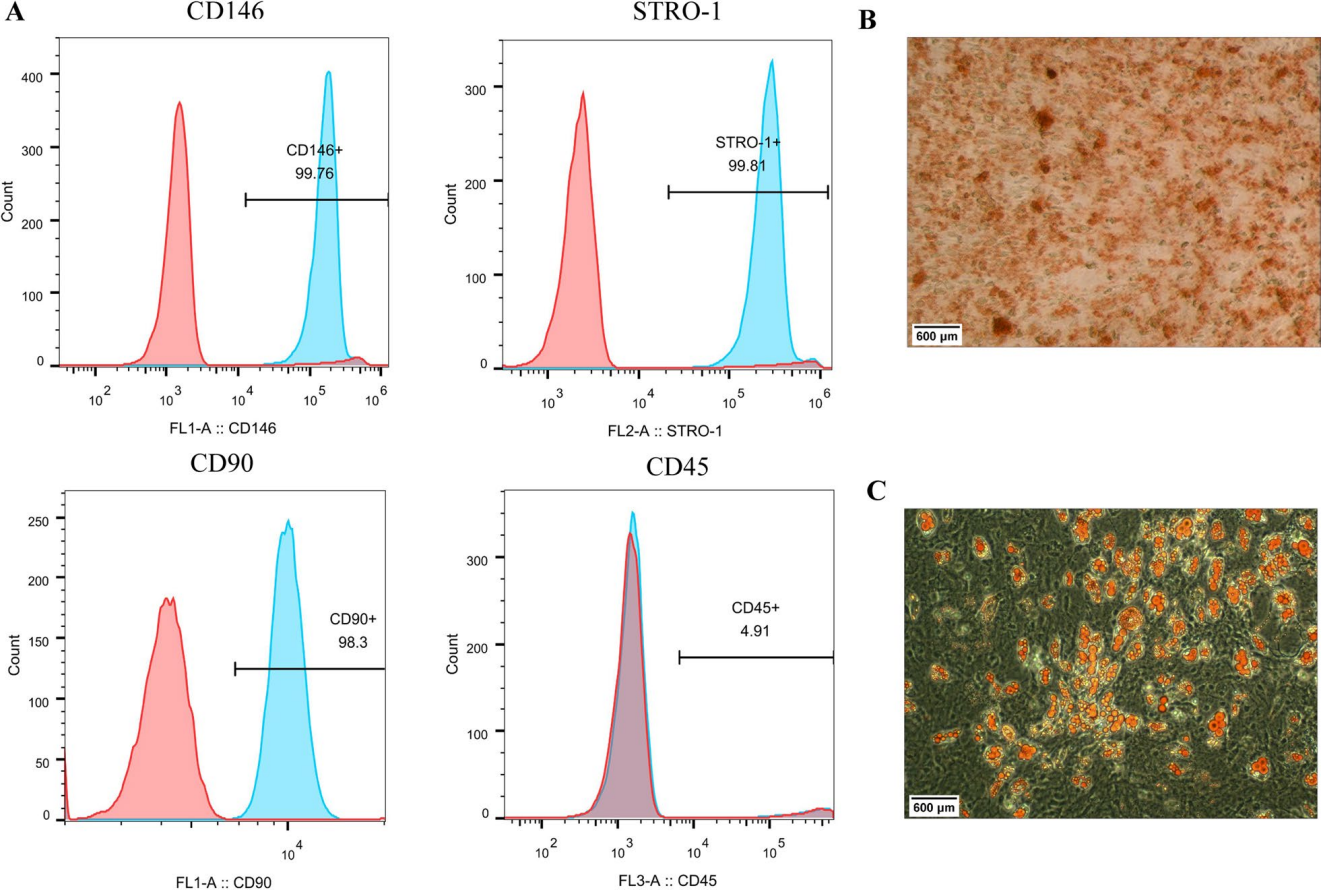
Isolation and identification of human periodontal ligament stem cells (hPDLSCs). **A** Expression of surface antigens CD146, CD90, STRO-1, and CD45 on hPDLSCs by flow cytometry; **B** Alizarin red staining for assessment of mineralized nodules formation in osteogenic differentiation of hPDLSCs; **C** Oil Red O staining for assessment of lipid droplet formation in adipogenic differentiation of hPDLSCs

To explore the role of oridonin in hPDLSCs, hPDLSCs were first isolated and then identified by flow cytometry and other methods. As results shown, hPDLSCs displayed positive expression for surface antigens CD146, CD90 and STRO-1, but negative expression for CD45 (Fig. 1A). Osteogenic and adipogenic induction was also performed on hPDLSCs, and there were mineralized nodules of osteogenic differentiation and lipid droplets of adipogenic differentiation in hPDLSCs cells observed by alizarin red staining and Oil Red O staining, respectively (Fig. 1B, C). These results suggested that hPDLSCs cells were successfully isolated in this study, so they were employed in subsequent experiments.

### Low-concentration oridonin is not cytotoxic to hPDLSCs

**Fig. 2:**
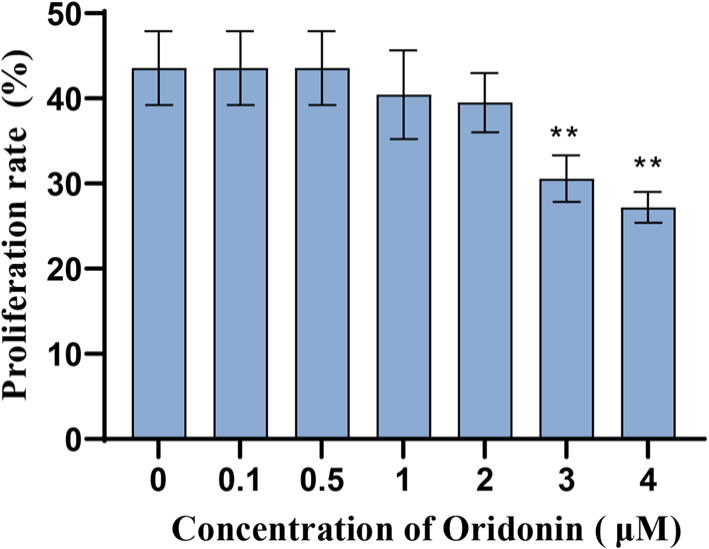
Low concentrations of oridonin were not cytotoxic to hPDLSCs. MTT was taken to detect the proliferation rate of hPDLSCs cells treated with oridonin at different concentrations (0, 0.1, 0.5, 1, 2, 3 and 4 μM), **P < 0.01 versus 0 concentrations

To clarify whether oridonin was cytotoxic to hPDLSCs, we cultured hPDLSCs with medium containing different concentrations (0, 0.1, 0.5, 1, 2, 3, and 4 μM) of oridonin for 24 h. And it was found that 0.1, 0.5, 1, and 2 μM of oridonin had no significant effect on the proliferation of hPDLSCs cells compared with the 0 μM group (*P* > 0.05), while the proliferation rate of hPDLSCs cells was notably reduced in the 3 μM and 4 μM groups (*P* < 0.05). The above suggested that 0–2 μM of oridonin was not significantly toxic to hPDLSCs cells (Fig. 2). Therefore, we chose 2 μM of oridonin to treat LPS-induced hPDLSCs cells.

### Oridonin attenuates the inhibition of proliferation, osteogenesis and adipogenic differentiation of hPDLSCs by lipopolysaccharide

**Fig. 3:**
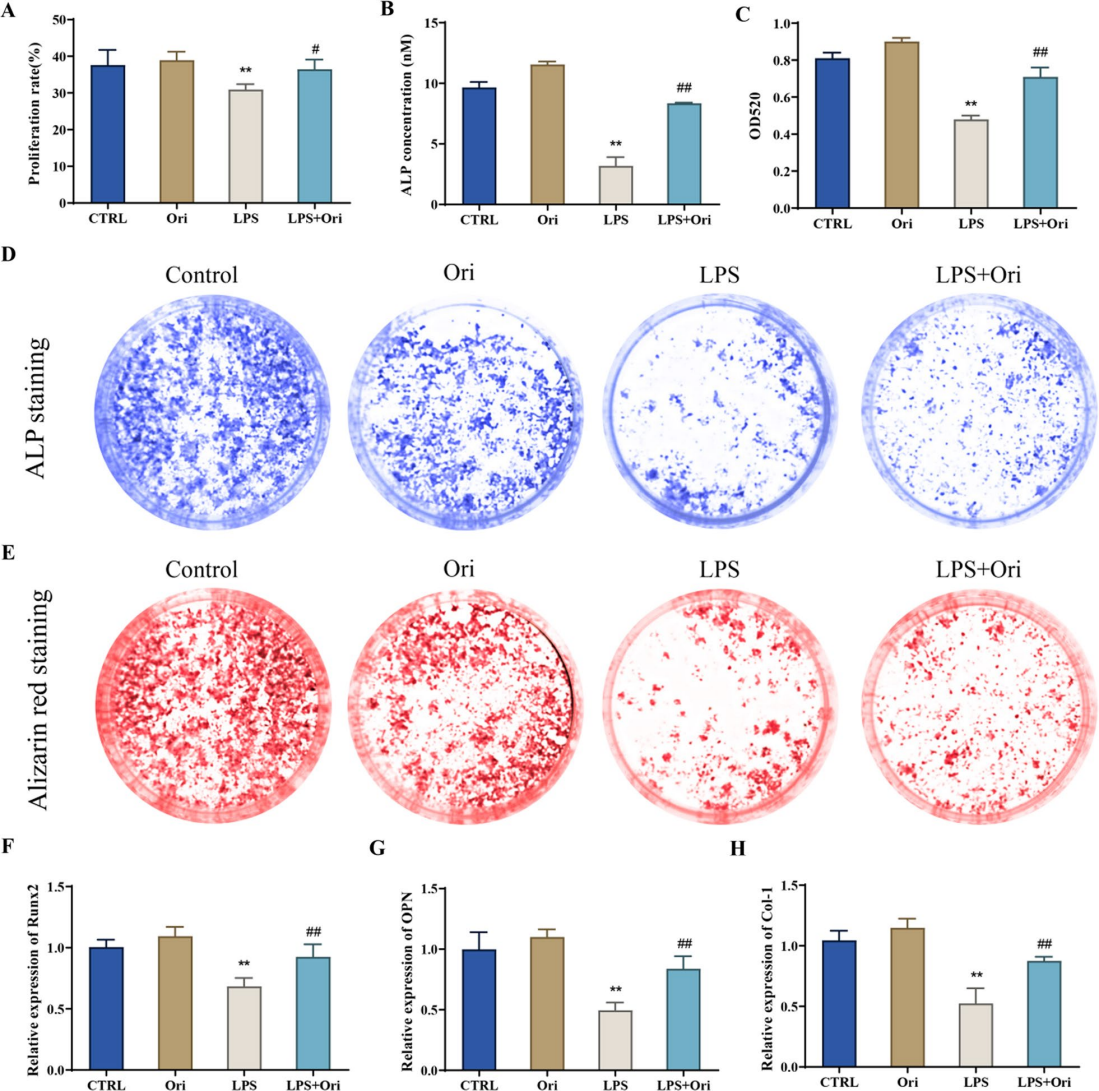
Oridonin alleviated the inhibitory effect of lipopolysaccharide (LPS) on the proliferation and osteogenic differentiation of hPDLSCs. **A** MTT to detect the proliferation rate of hPDLSCs cells in each group; B-D, ALP staining (**B**/**D**) and alizarin red staining (**C**/**E**) to assess ALP concentration and calcium nodule formation in hPDLSCs cells, respectively; **F**–**H**, qRT-PCR to detect the expression level of osteogenesis-related genes Runx2 (**F**), OPN (**G**) and Col-1 (**H**) in hPDLSCs cells in each group, **P < 0.01 versus CTRL group, #*P* < 0.05 and ##*P* < 0.01 versus LPS group

Subsequently, we explored the role of oridonin in inhibition of proliferation and osteogenic differentiation in LPS-induced hPDLSCs. The results reported that compared with the CTRL group, the proliferation rate, ALP concentration, calcium nodule formation level, and the expression level of osteogenesis-related genes (Runx2, OPN, Col-1) of hPDLSCs cells in the LPS group were markedly decreased (*P* < 0.01). While compared with the LPS group, the proliferation rate, ALP concentration, calcium nodule formation level, and the expression level of osteogenesis-related genes (Runx2, OPN, Col-1) of hPDLSCs cells in the LPS + Ori group were greatly increased (*P* < 0.05). There was no significant difference between the above phenotypes of hPDLSCs cells in the Ori group and those in the CTRL group (*P* > 0.05) (Fig. 3A–H). In summary, oridonin was able to significantly reduce LPS-induced inhibitory effects on hPDLSCs proliferation and osteogenic differentiation.

### Oridonin alleviates LPS-induced inflammation and endoplasmic reticulum stress in hPDLSCs

**Fig. 4:**
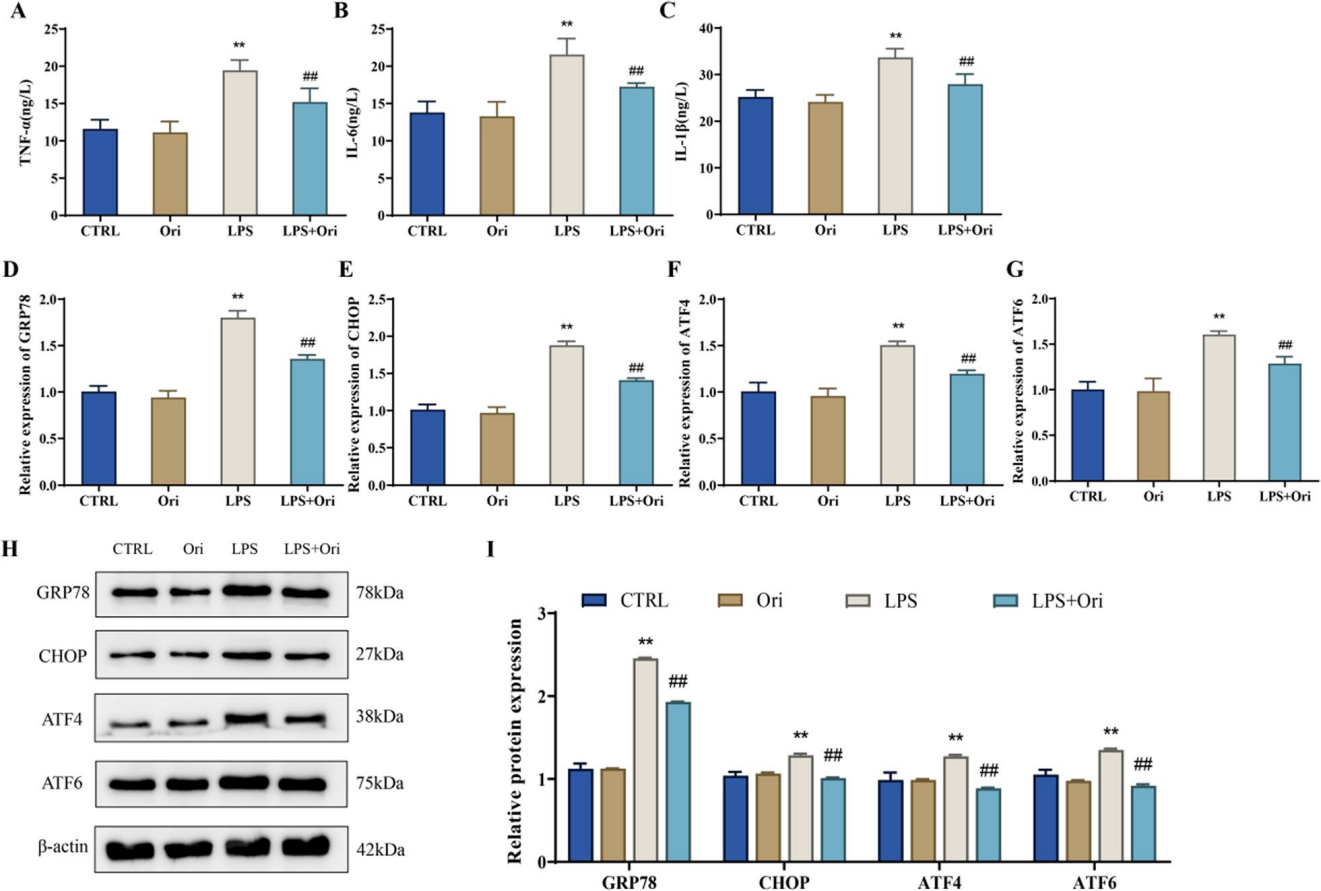
Oridonin attenuates inflammation and ER stress in LPS-induced hPDLSCs. **A**–**C**, the level of proinflammatory cytokines TNF-α (**A**), IL-6 (**B**) and IL-1β (**C**) in hPDLSCs cells of each group was measured by ELISA; **D**–**G**, the expression level of GRP78 (**D**), CHOP (**E**), ATF4 (**F**) and ATF6 (**G**) in hPDLSCs cells of each group was detected by qRT-PCR; **H**/**I**, the protein expression level of GRP78, CHOP, ATF4 and ATF6 in cells was tested by Western blot and the relative protein expression level was analyzed, **P < 0.01 versus CTRL group, ##*P* < 0.01 versus LPS group

We measured the levels of proinflammatory factors and the expression levels of ER stress-related genes in LPS-induced hPDLSCs cells. And it was revealed that the level of proinflammatory cytokines TNF-α, IL-1β, and IL-6 as well as the mRNA and protein expression level of ER stress-related genes GRP78, CHOP, ATF4, and ATF6 exhibited a noticeable climb in LPS-treated hPDLSCs compared with the CTRL group (*P* < 0.01). It suggested that LPS could induce inflammation and ER stress in hPDLSCs cells. However, 2 μM of oridonin remarkably lowered the TNF-α, IL-1β, and IL-6 levels together with the mRNA and protein expression level of GRP78, CHOP, ATF4, and ATF6 in hPDLSCs cells in LPS group (*P* < 0.01) (Fig. 4). The above indicated that oridonin alleviated ER stress in LPS-induced hPDLSCs by decreasing the expression of GRP78, CHOP, ATF4, and ATF6.

### Oridonin inhibits the NF-κB/NLRP3 pathway in LPS-induced hPDLSCs cells

**Fig. 5:**
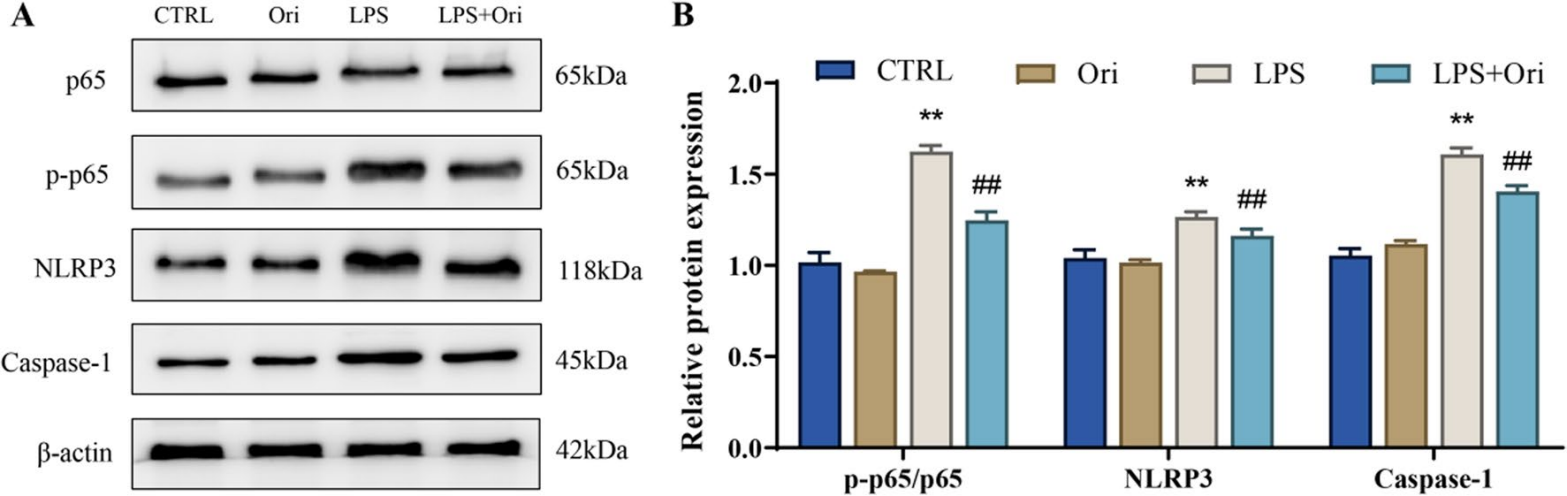
Oridonin inhibits NF-κB/NLRP3 pathway in LPS-induced hPDLSCs cells. A/B, The protein expression level of p65, p-p65, NLRP3 and Caspase-1 in hPDLSCs of each group were detected by Western blot (**A**), the p-p65/p65 ratio and the relative protein expression level of NLRP3 and Caspase-1 were analyzed (**B**), **P < 0.01 versus CTRL group, ##*P* < 0.01 versus LPS group

**Fig. 6:**
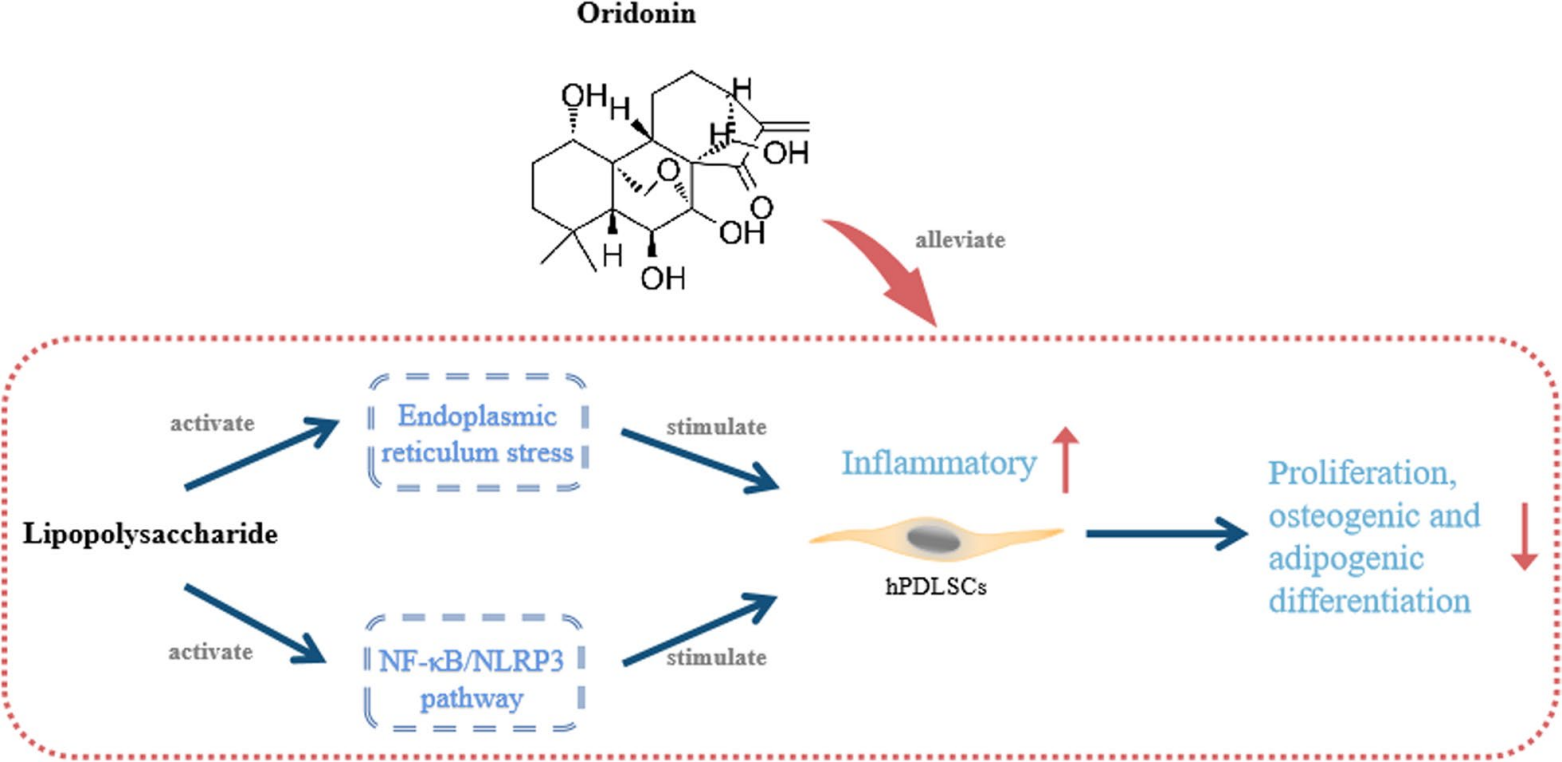
The schematic diagram shows the mechanism of LPS-induced cell injury in hPDLSCs regulated by oridonin. hPDLSCs, human periodontal ligament stem cells; LPS, lipopolysaccharide

Therefore, we speculated that oridonin may play a protective role by inhibiting the NF-κB/NLRP3 pathway in LPS-induced hPDLSCs cells, and then we measured the expression level of NF-κB/NLRP3 pathway-related proteins. The outcomes revealed that LPS treatment highly increased the protein expression level of p-p65, NLRP3 and Caspase-1 as well as the ratio of p-p65/p65 in hPDLSCs cells (*P* < 0.01); whereas 2 μM of oridonin considerably reduced the protein expression level of p-p65, NLRP3 and Caspase-1 as well as the ratio of p-p65/p65 in hPDLSCs cells in the LPS group (*P* < 0.01) (Fig. 5A, B). Taken together, these results indicated that oridonin promotes proliferation and osteogenic differentiation through inhibiting LPS-activated NF-κB/NLRP3 pathway activity in hPDLSCs cells (Fig. 6).

## Discussion

Periodontitis is mainly induced by microbial excitation and affects the tooth support structure [21]. hPDLSCs have been shown to have regenerative capacity in periodontal tissues [22]. Thanks to the multilineage differentiation potential and high periodontal correlation of hPDLSCs, hPDLSCs are the most reliable seed cells for periodontal tissue regeneration [4]. Nevertheless, the function of stem cells is often influenced by diseases such as aging and inflammation, and how to enhance the viability and osteogenic potential of hPDLSCs has become a hot topic for scholars [23, 24]. Periodontal tissue regeneration is a complex healing cascade caused by the coordinated interaction among stem cells, biomaterials, biological factors, and the host immune system [3]. In 2004, Seo et al. [4] first isolated and cultured hPDLSCs from periodontal ligament tissue. Subsequently, it has been confirmed that hPDLSCs can not only proliferate and clone, but also differentiate into different cell lineages such as adipoid cells, neuron-like cells, and osteoblast-like cells after appropriate induction [25]. In this study, hPDLSCs were isolated from the roots of human premolar teeth, and surface antigen detection revealed positive expression of CD146 and STRO-1 and negative expression of CD45. It could be observed from Alizarin red staining and Oil Red O staining, respectively, that after being induced, hPDLSCs produce mineralized nodules of osteogenic differentiation and lipid droplets of adipogenic differentiation, suggesting that hPDLSCs were successfully isolated and identified.

Studies have displayed that if hPDLSCs are placed in an inflammatory environment of periodontitis lesions for a long time, their repair and reconstruction abilities and stem cell characteristics will be affected, thus causing reduced repair ability of hPDLSCs [26]. LPS produced by Porphyromonas gingivalis has a significant cytotoxic effect on periodontal ligament cells. LPS, in turn, aggravates inflammation in periodontal tissues and destroys the homeostasis in the internal environment by promoting the synthesis and release of various inflammatory mediators by periodontal ligament cells. As a result, LPS plays a key role in the occurrence and development of periodontitis [27]. Enhanced alkaline phosphatase activity and calcium deposition are important markers of osteogenic differentiation in cells. Alkaline phosphatase appears in early osteoblast differentiation and can accelerate the mineralization of bone matrix [28]. And extracellular calcium deposition is a crucial manifestation in late osteogenesis, and cell mineralization ability can be visually assessed by alizarin red S staining and semi-quantitative analysis [29]. Also, the expression of osteoblast-related genes is an indicator for detecting the osteogenic differentiation ability of cells. Specifically, COL1, an early marker of osteogenic differentiation, can not only promote cell adhesion and osteoblast phenotype formation, but also provide a framework for mineral deposition [30]. RUNX2 is a species-specific transcription factor that induces cell osteoblastic differentiation and further regulates the maturation of osteoblasts [31]. OPN, a non-collagenous protein synthesized by osteoblasts, is a late marker of osteoblastic differentiation [32]. In this study, it was found that LPS decreased the ALP concentration, calcium deposition level, and the expression level of osteogenesis-related genes (Runx2, OPN, Col-1) of the cells. However, oridonin could significantly increase the ALP concentration, calcium deposition level, and the expression level of osteogenesis-related genes (Runx2, OPN, Col-1) in hPDLSCs cells inhibited by LPS. Consequently, the role of oridonin in the proliferation and osteogenic differentiation of hPDLSCs is further confirmed.

GRP78 is an ER chaperone, and the corresponding expression greatly went up during ER stress [33]. Specifically speaking, when ER stress occurs, ATF4 and ATF46 are separated from GRP78, simultaneously translocate to the Golgi, then act as transcription factors and bind to ERS-related response elements, resulting in the activation of the UPR, modifying or regulating the folding of proteins [34]. Besides, excessive or long ER stress leads to cell apoptosis, and CHOP is a specific mediator of the ER stress-induced apoptosis pathway [35]. TNF-α and IL-6 are multifunctional cytokines produced by monocytes, lymphocytes and fibroblasts, and have extensive biological activities. It has been found that LPS promotes IL-6 and TNF-α secretion from hPDLSCs [36, 37]. In this study, LPS markedly increased the TNF-α, IL-6, and IL-1β level and promoted ER stress in hPDLSCs cells, while oridonin treatment inhibited inflammatory response and ER stress in LPS-induced hPDLSCs cells.

The NLRP3 inflammasome is a multiprotein complex composed of NOD-like receptor, NLRP3, ASC and Caspase-1. And it mediates the processing and maturation of IL-1β so that IL-β can induce inflammation [38]. Oridonin, as results have shown, can target NLRP3 to exert an anti-inflammatory effect, thereby effectively inhibiting the progression of NLRP3-related diseases [14]. In addition, studies have reported that intraperitoneal injection of derivatives of oridonin at 5 mg/kg or 7.5 mg/kg in a mouse colitis model can improve colitis via reducing the expression of Th1/Th17, TNF-α, IFN-γ, and IL-17A and inhibiting the expression of NF-kB (p65), thereby improving colitis [39]. In this study, the protein expression levels of p-p65, NLRP3 and Caspase-1 in LPS group cells displayed a marked rise. In contrast, the protein levels of p-p65, NLRP3 and Caspase-1 in hPDLSCs cells exhibited a significant decrease after oridonin treatment. The above indicated that the mechanism of oridonin may function through the inhibition of the NF-κB/NLRP3 inflammasome signaling pathway. However, the correlation between NF-κB/NLRP3 pathway inhibition and oridonin's function of promoting proliferation, osteogenic differentiation and inhibiting inflammation and ER stress was not further explored experimentally in this study. Besides, the protective effect of oridonin in LPS-induced hPDLSCs cells has only been confirmed in in vitro experiments, and it remains unclear whether it has the same efficacy *invivo*. Therefore, more basic experiments are need to verify the above issues (Additional file 1).

## Conclusions

In summary, oridonin can not only promote the proliferation, osteogenic differentiation and adipogenic differentiation abilities of hPDLSCs, but also inhibit the ER stress and inflammatory response of cells. Besides, oridonin alleviates the effects of LPS on the proliferation, osteogenesis and adipogenic abilities of hPDLSCs possibly by inhibiting the inflammatory response, ER stress as well as the NF-κB/NLRP3 inflammasome signaling pathway. Oridonin has potential as a novel natural medicine for alleviating periodontitis.

## Supplementary Information

**Additional file 1**. All the original uncropped Western Blots in the article.

## Data Availability

The datasets used and/or analyzed during the current study are available from corresponding author on reasonable request.
