# Supplementary figures and images for: Oridonin alleviates the inhibitory effect of lipopolysaccharide on the proliferation and osteogenic potential of periodontal ligament stem cells by inhibiting endoplasmic reticulum stress and NF-κB/NLRP3 inflammasome signaling
Source: BMC Oral Health. 2023 Mar 9;23:137. doi: 10.1186/s12903-023-02827-0 (PMC9999511; doi:10.1186/s12903-023-02827-0)

**Figure 4H**


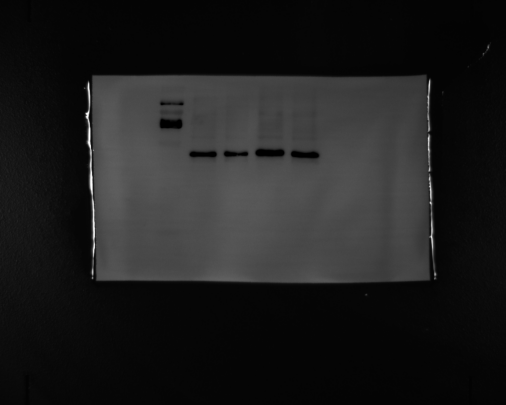


GRP78


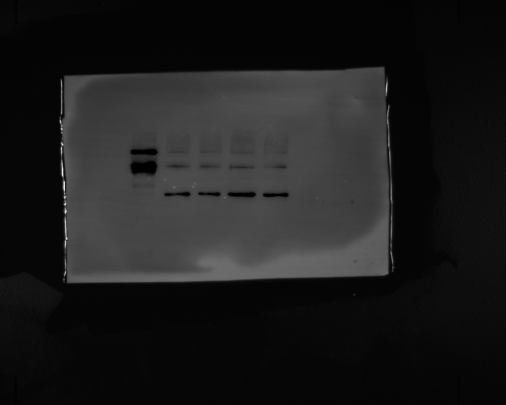


CHOP


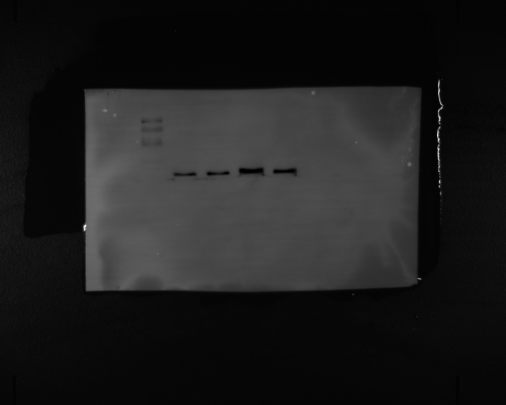


ATF4


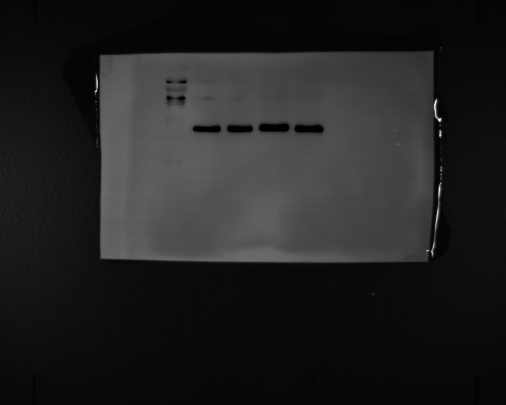


ATF6


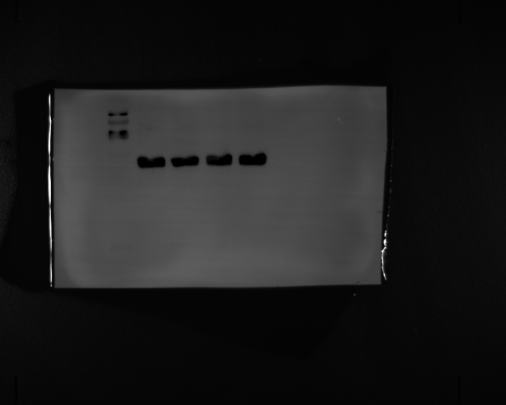


β-actin

**Figure 5A**


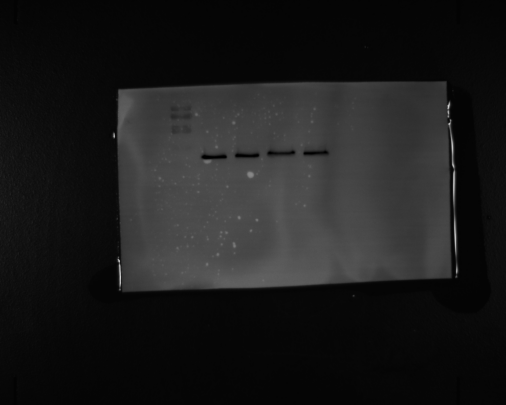


p65


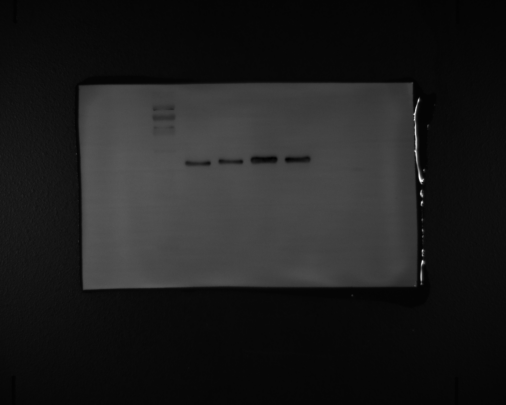


p-p65


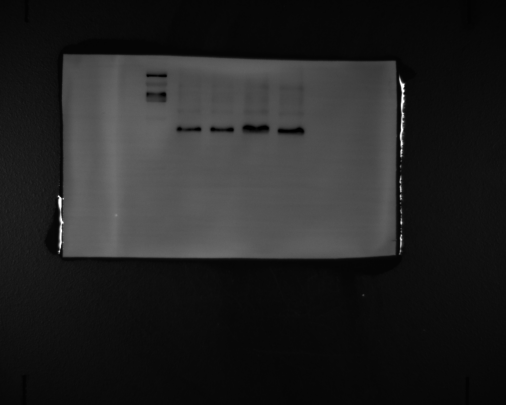


NLRP3


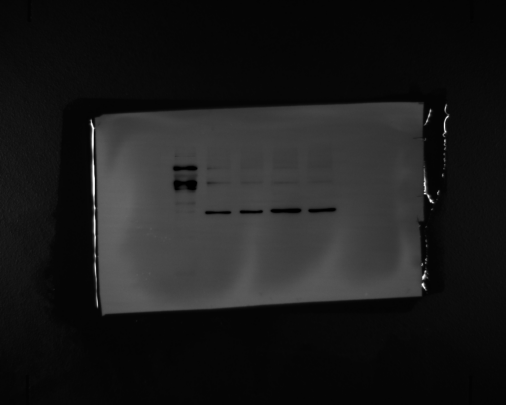


Caspase-1


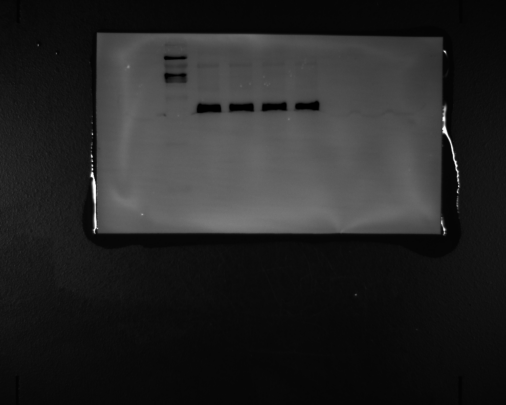


β-actin

Supplement: Supplementary file 1 — Additional file 1. All the original uncropped Western Blots in the article. [file 12903_2023_2827_MOESM1_ESM.docx]
